# Promotion Effect of Carboxymethyl Chitosan on Dental Caries via Intrafibrillar Mineralization of Collagen and Dentin Remineralization

**DOI:** 10.3390/ma15144835

**Published:** 2022-07-12

**Authors:** Qi Zhang, Jiaxin Guo, Zihua Huang, Sui Mai

**Affiliations:** 1 Hospital of Stomatology, Sun Yat-sen University, Guangzhou 510055, China; zhangq255@mail2.sysu.edu.cn (Q.Z.); guojx5@mail2.sysu.edu.cn (J.G.); 2 Guangdong Provincial Key Laboratory of Stomatology, Guangzhou 510080, China; 3 Institute of Stomatology, Sun Yat-sen University, Guangzhou 510080, China; hzh9320187@csu.edu.cn; 4 Department of Stomatology, Xiangya Stomatological Hospital, Central South University, Changsha 410008, China

**Keywords:** carboxymethyl chitosan, intrafibrillar mineralization, dentin remineralization, dentin bonding, bond durability

## Abstract

Objective: To observe ultrastructural changes during the process of carboxymethyl chitosan (CMC)-mediated intrafibrillar mineralization, we evaluated the biomimetic remineralization potential of CMC in type-I collagen fibrils and membranes, and further explored the bond strength as well as the bond interfacial integrity of the biomimetic remineralized artificial caries-affected dentin (ACAD). Methods: A mineralized solution containing 200 μg/mL CMC was used to induce type-I collagen biomimetic remineralization in ACAD, while traditional mineralization without CMC was used as a control. The process and pattern of mineralization were investigated by transmission electron microscopy (TEM), scanning electron microscopy (SEM), and energy-dispersive X-ray spectroscopy (EDS) as well as structured illumination microscopy (SIM). The Vickers hardness test was used to quantify the dentin hardness, while the microtensile bond strength (µTBS) test was used to assess the bond strength and durability. The bond interfacial integrity was evaluated by a confocal laser scanning microscope (CLSM). Results: TEM, SEM, and SIM images showed that CMC had a positive effect on stabilizing amorphous calcium phosphate (ACP) and promoting intrafibrillar mineralization, while extrafibrillar mineralization was formed without CMC. Furthermore, hardness evaluation and µTBS proved that CMC significantly increased dentin hardness and bond strength. CLSM indicated that CMC could create a significantly better bond interfacial integrity with less of a micro-gap in ACAD. Significance: CMC possessed the ability to promote intrafibrillar mineralization and remineralization in demineralized caries dentin lesions, as well as improve bond performance, which implied its potential in carious dentin demineralization or dentin hypersensitivity and possibly even as a possible material for indirect pulp-capping, to deal with deep caries. Highlights: CMC possessed the ability to induce intrafibrillar mineralization effectively; the bond strength and bond durability of demineralized caries dentin were improved via CMC-induced remineralization; the CMC-induced remineralization complex is a potential material for indirect pulp-capping, to deal with deep caries.

## 1. Introduction

Currently, the most prevalent oral disease is still dental caries in many developing and industrialized countries. It has been proven that the imbalance between demineralization and remineralization processes leads to the dynamic disease process of dental caries [1]. In general, carious dentin can be divided into two layers that are chemically and ultra-microscopically distinct. Since the outer layer (infected dentin) is irreversibly denatured and cannot be remineralized, it should be removed. However, the inner layer (caries-affected dentin, CAD) is reversible, is not infected, and can be remineralized [2,3]. Minimally invasive dentistry (MID) emphasizes the preservation of CAD and advocates maximizing the retention of tooth tissues during vital pulp therapy via remineralization [4].

Due to its weaker mechanical qualities, CAD has been shown to have a lower bonding strength than sound dentin [5]. When dentin is demineralized, matrix metalloproteinases (MMPs) are released to destroy exposed collagen fibrils, thereby decreasing the durability of resin–dentin adhesion. Inhibiting the activity of MMP can avoid collagen fibril degradation for a short time, but collagen fibrils can still be degraded by natural aging. Research has indicated that only mineralized collagen fibrils can stop the degradation caused by MMPs and aging, and can thus restore the hardness of natural mineralized dentin, remove the enzyme mechanism, and maintain the stability of the resin–dentin interface [6]. Therefore, the remineralization of demineralized dentin is critical for improving bonding stability and preventing primary and secondary cavities.

Dentin is composed of an inorganic component and an organic matrix, the majority of which is type-I collagen [7]. Remineralization in dentin is actually a process in which the inorganic minerals interact with the organic matrix. In fact, the abundant organic matrix in dentin makes it difficult to remineralize the remaining demineralized dentin. Generally speaking, dentin collagen mineralization can be split into two categories: extrafibrillar and intrafibrillar mineralization. The former occurs in the intervals between collagen fibrils, while the latter occurs in the gap zones, with the depositing minerals extending into the fibrils [8,9]. In nature, only intrafibrillar mineralization in collagen fibrils can arrange intrafibrillar minerals regularly and hierarchically, thereby resulting in a significantly optimized physical performance. How to stabilize amorphous calcium phosphate (ACP) and guide it into the 40 nm-long interstitial space of the collagen fibril is, therefore, the key to inducing intrafibrillar mineralization.

In the past few years, a biomimetic remineralizing strategy that mimics the natural biomineralization process has been put forward for remineralizing demineralized dentin in caries. Dentin matrix protein 1 (DMP-1), a natural non-collagen protein (NCP), is widely involved in the process of regulating dentin remineralization, with a structure of highly phosphorylated serine and threonine, and the ability to chelate calcium ions to stabilize ACP in solution to form amorphous nano-precursors [10]. Studies have shown that NCPs can be replaced with a biomimetic analog to initiate the remineralization process. Thus far, NCPs such as polyaspartic acid (PASP) and polyacrylic acid (PAA), which have rich carboxyl groups, have been proven to form amorphous nano-precursors by stabilizing ACP [11,12].

Carboxymethyl chitosan (CMC) is rich in carboxyl groups and studies have shown that it is able to decelerate or even inhibit the spontaneous precipitation of calcium phosphate [13]. Therefore, water-soluble CMC might take advantage of its chelating capacity to assist the formation of ACP nano-precursors [14]. Our previous studies preliminarily investigated the function of CMC in CAD biomimetic remineralization and validated the combined effects of CMC molecular weights and concentrations on the collagen mineralization pattern and its extent by regulating the stability of CMC-ACP precursors [15,16].

A change in the diameter and transverse striation of collagen fibrils before and after mineralization can be observed by electron microscopy, but it cannot determine whether mineralization occurs inside or outside the collagen fibrils. Therefore, in addition to finding a better way to stabilize ACP, verifying the occurrence of intrafibrillar mineralization is a difficult task in mineralization research.

In the present study, we established two-dimensional (2D) and three-dimensional (3D) models of collagen, as well as models of CAD. Then, we validated the role of CMC as an analog of NCP and investigated its efficiency and impact on the biomimetic mineralization and demineralization of dentin, aiming to provide potential therapeutic strategies for clinical caries treatment. In addition to TEM and SEM, we also used SIM to observe the three-dimensional positional relationship between hydroxyapatite and collagen, to confirm whether intrafibrillar remineralization was occurring. In addition, we assumed a null hypothesis that CMC could neither guide intrafibrillar mineralization in collagen fibrils nor enhance the bonding action of CAD.

## 2. Materials and Methods

### 2.1. Mineralizing Media Preparation

A hydrophilic resin matrix containing 70 weight percent of bisphenol A diglycidyl ether dimethacrylate (Bis-GMA, Esstech, Essington, PA, USA), 28.75 weight percent 2-hydroxyethyl methacrylate (HEMA, Esstech, Essington, PA, USA), 1 weight percent ethyl N,N-dimethyl-4-aminobenzoate(EDMAB, Esstech, Essington, PA, USA), 0.25 weight percent camphorquinone (CQ, Esstech, Essington, PA, USA), and 17.6 weight percent white Portland cement powder (Lehigh Cement Company, Allentown, PA, USA) were used to make composite resin disks [15]. Amorphous calcium phosphate (ACP) was converted to carbonated apatite with the help of calcium hydroxide (pH > 9.25) in Portland cement. The phosphate ions come from phosphate-buffered saline (PBS).

**Figure 1:**
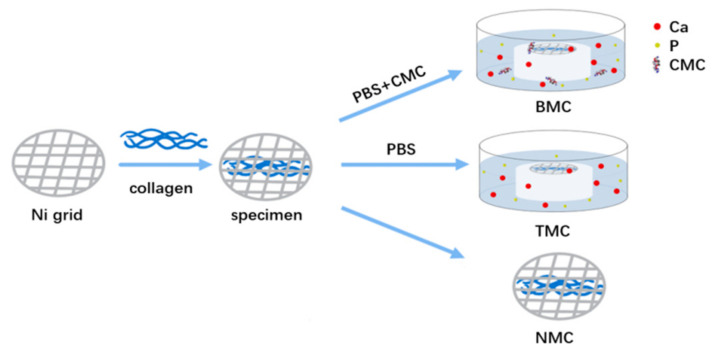
Schematic representation of collagen specimen fabrication.

In the biomimetic mineralized collagen (BMC) group, collagen specimens were placed over a composite disk while floating upside-down in PBS (pH = 7.4) solution containing 200 μg/mL CMC (Mw: 150 kDa, RuibioC3105, Germany) in a 100% humidity chamber. As for the traditional mineralized collagen (TMC), the collagen specimens were placed on a composite disk while floating upside-down over PBS solution without CMC in a 100% humidity chamber. The non-mineralized collagen (NMC) worked as a negative control without additional processing (Figure 1).

### 2.2. Collagen Fibril Mineralization

#### 2.2.1. TEM of Mineralized Reconstituted Single-Layer Collagen Fibrils

Collagen stock solution was prepared by dissolving lyophilized type-I collagen powder (Sigma-Aldrich, St. Louis, MO, USA), derived from rat tail tendons, in 0.1 M acetic acid (pH 3.0) at 4 °C overnight. To reconstitute a single layer of type-I collagen fibrils, a 0.1 mg/mL collagen stock solution was neutralized with 1 percent ammonia vapor for 2 h on formvar-and-carbon-coated 400-mesh Ni TEM grids (Electron Microscopy Sciences, Hatfield, PA, USA). The collagen-coated grids were then treated for 4 h with 0.3 M 1-ethyl-3-(3-dimethylaminopropyl)-carbodiimide (EDC, Thermo Scientific Pierce, Rockford, IL, USA)/0.06 M N-hydroxy succinimide (NHS, Thermo Scientific Pierce, Rockford, IL, USA) to promote collagen cross-linking and, thus, stabilize the collagen structure. The grids were then mineralized for 24, 48, and 72 h using the biomimetic and traditional mineralized solution described above. After that, grid samples were characterized using a transmission electron microscopy TEM (TEM 1400; JEOL, Tokyo, Japan) with a 120 kV accelerating voltage.

#### 2.2.2. SEM and EDS of Mineralized Three-Dimensional Type-I Collagen Membranes

A collagen membrane composed of reconstituted bovine type-I collagen (Heal-all, Yantai Zhenghai Biotechnology Co., Yantai, China) was purchased for use as the three-dimensional collagen matrix. The commercial collagen membranes were mineralized in the biomimetic and traditional mineralized solution for 24 h. Before observations, all specimens were sputter-coated with gold. A field-emission scanning electron microscope (SEM, Quanta 400 FEG, FEI, Paris, France) with an accelerating voltage of 20.0 kV was used to observe the morphologies of the membrane surfaces. The elemental analysis of Ca and P were collected by energy-dispersive X-ray spectroscopy (EDS) in area analysis mode. The operating parameters were: 20.0 kV accelerating voltage, a Si (Li) detector, and an image size of 80 μm × 80 μm.

#### 2.2.3. Spatial Distribution of the ACP within the Collagen Fibrils

Laser confocal culture dishes (LCCDs) were pretreated with a total of 200 μL of 3-aminopropyl triethoxysilane APTES (5 *v*/*v*% ethanol solution) [17]. After cleaning with absolute ethanol and deionized water, LCCDs were dried at 100 °C. The modified LCCDs were subsequently cross-linked with 0.05 weight percent glutaraldehyde for 2 h after being incubated with a total of 200 μL type-I collagen solution (100 g/mL) for 12 h.

The collagen fibrils attached to the LCCDs were labeled via immunofluorescent staining. The type-I collagen fibrils were dissolved in blocking buffer (Solarbio Science, Beijing, China) at 37 °C for 1 h. After that, the collagen fibrils were incubated with 200 µL of rabbit anti-mouse antibody (1:50; AF7001, Affinity, Changzhou, China) at 4 °C overnight and then cultured for 2 h at room temperature with goat anti-mouse IgG (H + L) CY3-conjugated (1:100; S0012, Affinity, China) while avoiding the light. After rinsing off the residual fluorescent reagents, the fluorescence-labeled collagen fibrils were set aside for further study.

The pretreated LCCDs with fluorescence-labeled collagen fibrils were mineralized in the as-prepared biomimetic and traditional mineralized solution for 24 h and were then labeled using 2 mL of 10 μM calcein for 20 min, to specifically bind with hydroxyapatite (HAP). Finally, 50 μL of ProLong gold antifade reagents (P10144, Thermo Fisher Scientific, Shanghai, China) were dropped on the mineralized labeled collagen fibrils, to provide antifade protection for the fluorochromes.

Confocal microscopy and structured illumination microscopy (SIM) imaging acquisitions were achieved by virtue of a Nikon-Structured Illumination Microscope (Nikon, Tokyo, Japan). SIM images were captured using a continuous activation mode, using continuous-frame-of-activation laser illumination (488 nm laser), followed by five frames of imaging laser illumination (561 nm laser). Then, the images were reconstructed using Nikon NIS Elements 5.21 software.

### 2.3. Mineralization and Characterization of the Artificial Caries-Affected Dentin (ACAD) Model

#### 2.3.1. Preparation and Mineralization of the ACAD model

An ACAD model was created followed by the method of Joves et al. [18]. In total, 132 extracted non-carious human third molars were obtained from patients aged 18–40 years old and were used under a protocol approved by the Sun Yat-sen University Ethics Committee.

Prior to use, the molars were kept in 0.5 percent chloramine T at 4 °C, for 1 month at most. To obtain CAD, the flat mid-coronal dentin surfaces were first exposed with a hard-tissue cutting machine (Accutom-50; Struers, Copenhagen, Denmark), which was equipped with a low-speed, water-cooled diamond wafering saw (330-CA RS-70300; Struers, Denmark). Clinically relevant smear layers were generated with a water-cooled polishing machine (Labopol-4; Struers, Denmark) equipped with 360-grit silicon carbide (SiC) abrasive paper, while demineralized specimens were generated by pH-cycling. The pH-cycling approach entails immersing the specimen first in a demineralization solution (containing 2.2 mM CaCl_2_, 2.2 mM NaH_2_PO_4_, 50 mM CH_3_COOH, pH 4.8) for 0.5 h and then in a solution containing 1.5 mM CaCl_2_, 0.9 mM NaH_2_PO_4_, and 0.15 mM KCl (pH 7.0) for 2.5 h for each of the 50 cycles [19].

The ACAD specimens were divided into 3 groups at random. In the biomimetic mineralized dentin (BMD) group, specimens were mineralized in the PBS solution containing composite silicate resin and 200 μg/mL CMC for 1 month. In the traditional mineralized dentin (TMD) group, specimens were mineralized in the PBS solution containing composite silicate resin without CMC for 1 month. In the demineralized dentin (DD) group, specimens were left untreated after the pH-cycling procedure. The remaining specimens without demineralization were the natural dentin (ND).

#### 2.3.2. Microhardness Test

For the microhardness test, 10 teeth from the BMD, TMD, DD, and ND groups were prepared. The Vickers hardness test was carried out on the surface of dentin specimens, using a standard Vickers indenter and a static load of 1000 g for 15 s [15], using a microhardness tester (DuraScan-20; Struers, Denmark).

#### 2.3.3. Microtensile Bond Strength (μTBS) Test and Failure Mode Analysis

Single Bond Universal (3M, ESPE) was used to bond the teeth from BMD, TMD, DD, and ND groups (n = 20). Half were bonded through an etch-and-rinse system and the other half were bonded in self-etch mode. Following that, four layers of 1 mm-thick composite resin (Z350, 3M, ESPE) were applied and then light-cured for 40 s. Subsequently, the samples were formed into bonded sticks measuring 0.9 × 0.9 × 8 mm^3^ using a hard-tissue microtome, after being stored in artificial saliva (AS) for 24 h and 10,000 thermal cycles (35 °C for 30 s, 15 °C for 2 s, 35 °C for 30 s, 45 °C for 2 s), based on the method used by Morresi et al. [20]. The microtensile bond strength (μTBS) was tested using a linear actuator (SMAC Europe Ltd., Horsham, West Sussex, UK). A stereoscopic microscope (Axio Observer Z1, Zeiss, Germany) was used to obtain the mode of the tensile fracture surface.

#### 2.3.4. Confocal Laser Scanning Microscope (CLSM)

After administering 37 percent H_3_PO_4_ for 15 s, three teeth from each dentin group were bonded with Single Bond Universal, mixed with 0.2 weight percent rhodamine B (Sigma-Aldrich, USA). All specimens were kept in the dark for 24 h before being sliced open and filled with 0.1 weight percent sodium fluorescein solutions (Sigma-Aldrich, USA) for 3 h. After that, the stained specimens were cut into 1.5 mm slices along the dental long axis and then polished for 1 min with 1200-grit SiC paper. Finally, a confocal laser scanning microscope (DM IRE2 CISM; Leica, Heidelberg, Germany) was used to view fluorescein at 488 nm and rhodamine B at 568 nm.

### 2.4. Statistical Analysis

SPSS 13.0 was used for the statistical analysis. The statistical significance level was pre-determined to be α = 0.05. After checking the data normality and equal variance assumptions, the microhardness [21] and μTBS [22] results were analyzed with one-way ANOVA and LSD-T tests.

## 3. Results

### 3.1. Effect of CMC as a Biomimetic Analog through 2D and 3D Collagen Models

#### 3.1.1. The Mineralization Mode of Single-Layer Mineralized Collagen Fibrils

**Figure 2:**
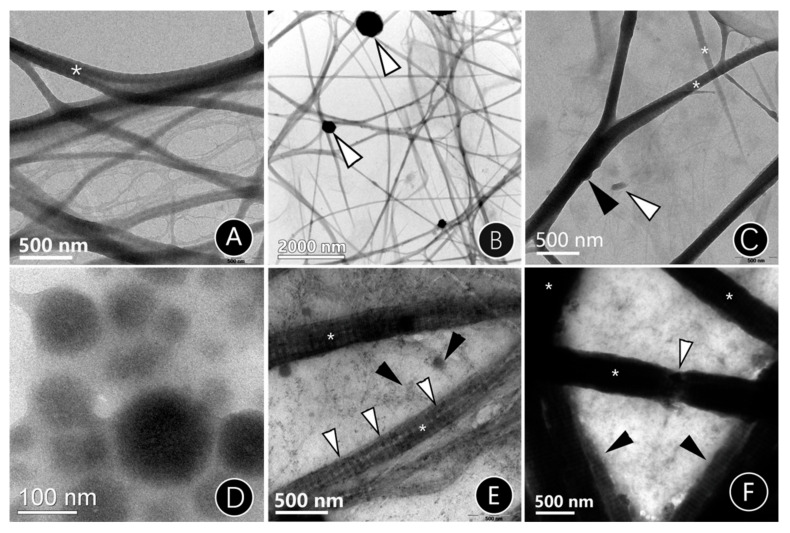
Unstained TEM images of single-layer mineralized collagen fibrils reconstituted on TEM grids. (**A**) The non-mineralized collagen (NMC) fibrils. Asterisk: collagen fibrils. (**B**) The traditional mineralized collagen (TMC), mineralized for 24 h. Spherical crystals could be identified on the collagen fibrils (white arrows). (**C**) The biomimetic mineralized collagen (BMC), mineralized for 24 h. White arrow: mineralized crystal deposited outside collagen fibers. Black arrow: mineralized crystal accumulated on the collagen fibers. (**D**) Nanoparticle size of CMC-ACP solutions for 24 h. (**E**) BMC, mineralized for 48 h. White arrows: mineralization, located in the dark zone. Black arrows: cluster mineralization between the collagen fibrils. (**F**) BMC, mineralized for 72 h. Asterisk: mineralized collagen fibrils. White arrow: Collagen fragmentation. Black arrows: cluster mineralization between the collagen fibrils.

A two-dimensional collagen mineralization model was used to examine the ability of CMC-ACP to mineralize collagen fibrils. Figure 2 shows the respective TEM images of calcium phosphate transformation in different mineralizing solutions. The TEM images indicated that apatite clusters were deposited extrafibrillarly when treated with a traditional mineralized solution for 24 h (Figure 2B, compared with the non-mineralized collagen (NMC) (Figure 2A). When treated with biomimetic mineralized solution, the unstained collagen fibrils became much darker; the intrafibrillar minerals transitioned into a crystalline state from an amorphous state over short time periods (Figure 2C,E,F). When mineralized for 24 h, the accumulation of CMC-ACP complexes appeared on the collage surface (Figure 2C, arrows). CMC-stabilized amorphous calcium phosphate (ACP) complexes could be observed as nanoparticles, with a particle diameter of around 150 nm (Figure 2D). As time increased to 48 h, infiltration with ACP could be observed in the fibrils, which induced the increased intensity of gap zones (Figure 2E). As ACP infiltrated the fibrils and transformed into apatite, the deposition of an intrafibrillar mineral-induced distinctive cross-band disappeared and band intensities in the overlap and gap zone increased (Figure 2F). In addition, fractures could be seen on these fibers, implying that the fibers had become hard and brittle due to the apatite crystals (Figure 2F, white arrow).

#### 3.1.2. Surface Microtopography of Three-Dimensional Collagen

**Figure 3:**
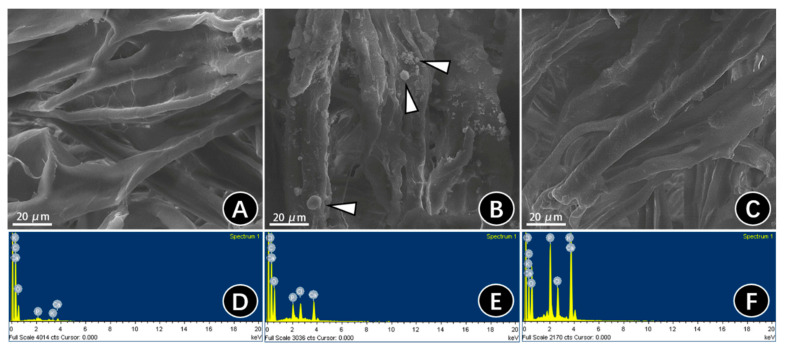
SEM micrographs and EDS analysis of the surface of three-dimensional collagen membranes treated for 72 h. SEM showing the (**A**) surface and (**D**) EDS plot of NMC. SEM showing the (**B**) surface and (**E**) corresponding EDS of TMC. Apatite clusters are visible, deposited on the surface of collagen (white arrows). SEM showing the (**C**) surface and (**F**) EDS plot of BMC.

Corresponding to the TEM of the 2D mineralized collagen model, the micromorphology of 3D mineralized collagen membranes was observed. Compared with NMC, which showed that the collagen possessed a smooth surface and uniform diameter (Figure 3A), apparent extrafibrillar mineralization was obtained in TMC, showing spherulitic clusters randomly distributed on the surface of collagen fibrils (Figure 3B arrows). Increasing diameters could be observed in BMC collagen with no extrafibrillar minerals or obvious banding pattern on the surface, indicating the intrafibrillar mineralization of collagen fibrils (Figure 3C).

EDS was used to verify whether the mineral (calcium and phosphorus elements) existed in the SEM images. We conducted area analysis and found that the EDS results of the NMC specimens confirmed the lack of detectable mineral elements (Figure 3D). A small number of minerals were detected in the TMC specimens, which may be because of the HA clusters (Figure 3E). Instead, the BMC specimens showed the highest amounts of calcium and phosphorus elements (Figure 3F), suggesting that BMC possessed a higher degree of mineralization than NMC and TMC. Furthermore, both the TMC and BMC groups showed similar ratios of Ca/P, which was closed to HA (Ca/P = 1.67).

#### 3.1.3. Location of Mineralization in Collagen Fibrils

**Figure 4:**
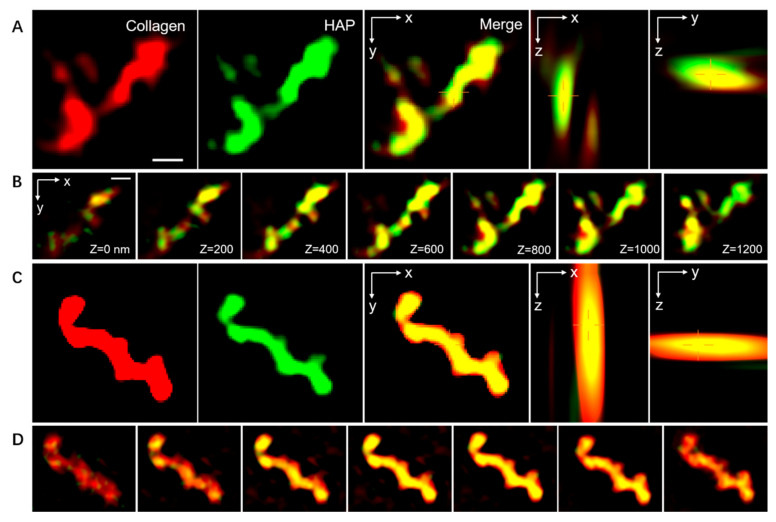
SIM micrographs of the mineralized type-I collagen fibrils. The collagen was tagged by Cy3 and emitted red fluorescence. The HAP was labeled with calcein, showing green fluorescence. The combination of red and green is shown in yellow. (**A**,**C**) The three-dimensional SIM images of the TMC and BMC. (**B**,**D**) The z-slices indicate the location of the HAP and collagen fibrils corresponding with (**A**,**C**). Scale bar: 500 nm.

In order to further confirm intrafibrillar mineralization, we used SIM to clarify the position relationship between the mineral HAP and collagen fibers. HAP molecules were mostly observed to have entered into the intrafibrillar space, while calcein-labeled calcium ions were found within the collagen from the three-dimensional visualization of the BMC group, indicating intrafibrillar mineralization (Figure 4C,D), while the calcein-labeled calcium was surrounded by the collagen fibrils, implying extrafibrillar mineralization in the TMC group (Figure 4A,B).

### 3.2. CMC-Induced Dentin Remineralization Improves Dentin Bonding and Adhesive Durability

#### 3.2.1. Hardness Evaluation

The dentin hardness values of each group are plotted in Table 1. The Vickers hardness of natural dentin (ND) was 75.1 ± 3.2 MPa. After demineralization, the hardness decreased to 55.2 ± 2.9 Mpa in the demineralized dentin (DD) group. When treated with biomimetic mineralized solution, the demineralized dentin was remineralized and obtained a hardness of 68.5 ± 2.5 MPa in the biomimetic mineralized dentin (BMD) group. The BMD group showed higher hardness values than the DD and conventional mineralized dentin (TMD) groups (*p* < 0.05), although it had lower values than the ND group (*p* < 0.05). The TMD group’s hardness values were not significantly different from those of the DD group (*p* > 0.05).

#### 3.2.2. Microtensile Bond Strength (µTBS) and Failure Analysis

Table 2 shows the means and standard deviations of TBS for each group. The µTBS decreased after thermal heat cycling in all treated groups except the BMC group, with more statistically significant results (*p* < 0.05) than before. The immediate bond strength of the BMD group (33.6 ± 4.3 MPa) was similar to that of the ND group (35.9 ± 7.0 MPa) (*p* < 0.05) but was much higher than that of the DD group (26.4 ± 5.9 MPa) (*p* < 0.05). After 10,000 thermal cycles, the µTBS values in the BMD group (26.7 ± 9.1 MPa) were substantially higher than those in the DD (2.5 ± 2.2 MPa) and TMD groups (1.9 ± 2.4) (*p* < 0.05), with no significant differences (*p* > 0.05), compared to those observed before cycling occurred. Conversely, the µTBS values in the DD (2.5 ± 2.2 MPa) and TMD groups (1.9 ± 2.4 MPa) were considerably lower than those observed before thermal cycling (*p* < 0.05).

**Figure 5:**
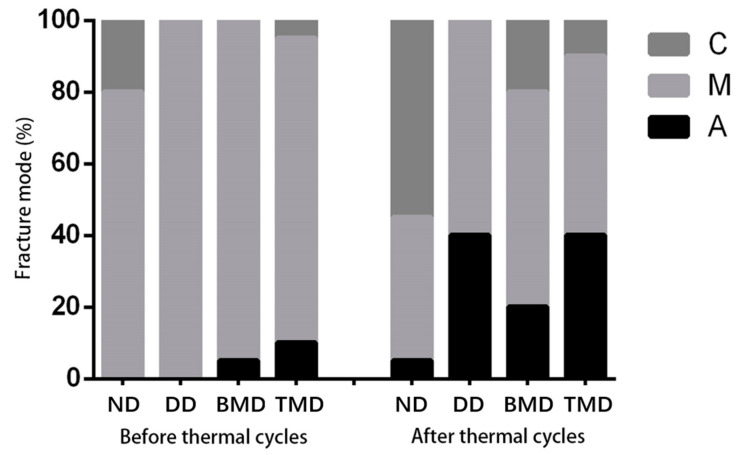
The fracture mode of dentin adhesive specimens before and after thermal cycling. C: Cohesive fracture. M: Mixed fracture. A: Adhesive layer fracture.

All groups were associated predominantly with adhesive failures; the failure mode is shown in Figure 5. Before thermal cycling, the incidences of mixed fractures were highest in each group, according to the fracture mode analysis. After 10,000 thermal cycles, the fracture mode of the adhesive layer increased (40%) in the DD group and TMD group, while that in the BMD group was relatively low (20%) but was still higher than that in the ND group (5%).

#### 3.2.3. CMC-Induced Remineralization Influence on the Properties of the Resin–Dentin Adhesive Interfacial Microenvironment

**Figure 6:**
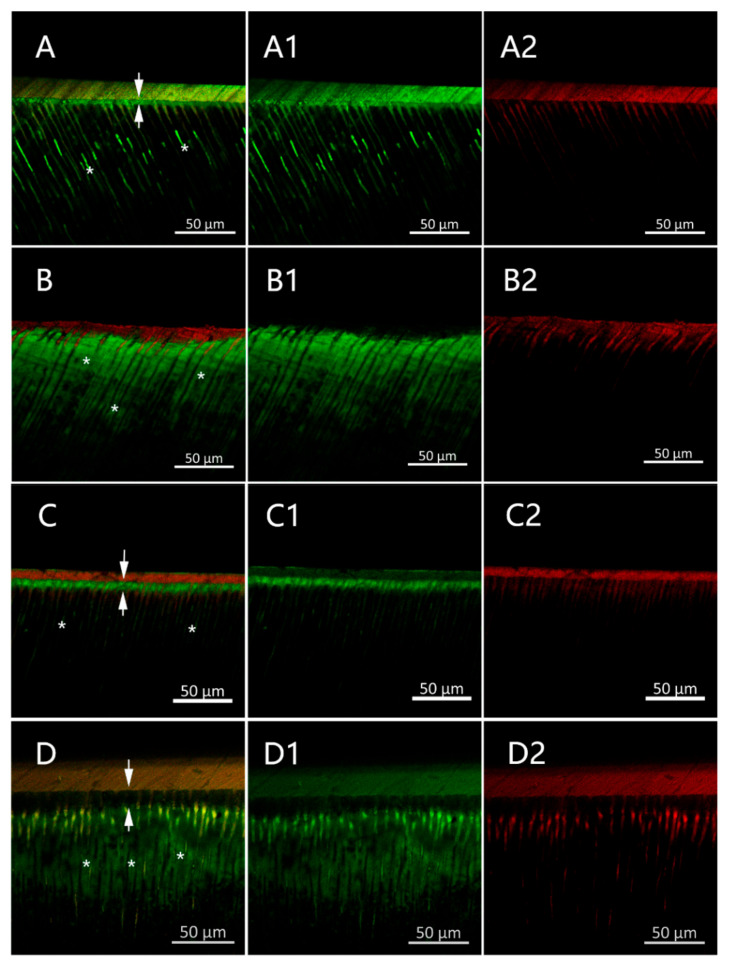
CLSM images of the resin–dentin interfaces in the ND (**A**), DD (**B**), BMD (**C**), and TMD (**D**) groups. X1: fluorescein excitation mode, X2: rhodamine excitation mode (X = **A**,**B**,**C**,**D**). Arrows: green fluorescent band. *: dentinal tubule.

The adhesive permeability was estimated via CLSM (Figure 6). Adhesive layers labeled by rhodamine B showed red fluorescence, while the sodium fluorescein-labeled permeating fluid showed green fluorescence. In the ND group, a 3–4 μm continuous green fluorescent band (Figure 6A, arrows) was observed, since the sodium fluorescein solution passed through the adhesive layer and permeated the dentinal tubules. The TMD group (Figure 6D) and ND group (Figure 6B) were similar, with the fluid permeating to approximately the same depth in the intertubular dentin. The dentin permeability in the BMD group was much lower than that in other groups, and the green fluorescein was mainly observed in the dentinal tubules, presenting a 6–7 μm highly permeating fluorescence band (Figure 6C, arrows), while a weaker fluorescence band was found at the surface of the TMD group (Figure 6D, arrows). Clearly, CMC-induced remineralization improved the infiltration of the hydrophobic adhesive macromonomers in the demineralized dentin matrix by reconstructing the interfacial microenvironment.

## 4. Discussion

Nowadays, synthesizing new biomimetic materials that can reconstruct dental structures by promoting remineralization is an increasingly important research direction in dental hard-tissue repair [23,24]. Thus, biomimetic analogs have been used for remineralization in ACAD [19,25]. In the present study, we explored the effect and primary mechanism of CMC-induced intrafibrillar mineralization through single-layer collagen fibril and three-dimensional collagen membrane models.

Collagen fibrils, derived from rat tails, displayed distinctive 67-nm cross-band characteristics after self-assembly, similar to the natural dental collagen. However, since the HA crystals generated in this fashion are too large in size compared to the gap zones of collagen fibrils, intrafibrillar remineralization cannot occur in a typical mineral solution containing only calcium and phosphate ions. Therefore, CMC can stabilize minerals such as calcium and phosphorus ions, which resulted in liquid ACP nanoparticles in the BMC mineralization system [26]. Collagen can serve as the primary template for attracting CMC-stabilized liquid ACP nanoparticles from the solution. The CMC/ACP nanocomplexes are then attracted to the collagen interstices by fluidic capillary forces, the interactions between the positive domains of collagen fibrils, and the negative charge of nanocomplexes to complete the intrafibrillar mineralization [27]. The above characterizations can contribute to the formation of high-speed and complete intrafibrillar mineralization.

The ability of CMC to stabilize ACP nanoparticles was observed by the TEM of grid-loaded collagen at different times. ACP particles that were stabilized by CMC began to encapsulate the collagen fibers and slowly infiltrated within 24 h. Then, ACP was clustered and the diameter was greatly reduced and transformed into HA kinetics at 48 h. The collagen fiber was further thickened after 72 h of mineralization and fractures were detected on the fibrils using TEM, indicating that the ACP in the collagen had changed into HA during this period, and mineral extensional growth had occurred outside the collagen fiber. The CMC-induced biomimetic mineralization of collagen fibers in this experiment was fast and complete. Likewise, the SEM and EDS images presented mineralization in both the BMC and TMC groups. In the TMC group, the crystals were so large that they could only be located on the surface of the membranes. However, in the BMC group, no obvious deposits could be seen on the surface, while the EDS analysis showed increased calcium deposition, which suggested the occurrence of intrafibrillar mineralization.

Structured illumination microscopy (SIM) was further conducted on the mineralized collagen fibrils to determine the exact spatial relationship between the remineralized particles and the collagen fibrils. The 3D resolution clearly revealed the spatial distribution between the organic components and the mineral by means of a 3D model [28,29]. The fluorescent molecules labeled on the collagen and HAP were activated randomly and captured individually. Then, by fitting using a model with subdiffractional accuracy, the location of each light point was determined. Based on the semi-permeable characteristics of collagen and the size exclusion theory, ACP/CMC nanocomposites were expected to diffuse into the intrafibrillar spaces owing to their small size (29). During the sample preparation for SIM, collagen fibrils were first labeled with Cy3; the HAP was then tagged with calcein after mineralization. The resulting two-dimensional images indicated that ACP infiltrated and was converted into HAP in the intrafibrillar spaces. Moreover, the 3D SIM images provided direct evidence that calcein-tagged HAP infiltrated into most of the intrafibrillar spaces and was homogeneously dispersed in the collagen fibrils, implying that ACP could eventually transform into the thermostatically stable HAP within the collagen fibrils, to provide excellent mechanical properties and collagen protection. These results demonstrated that CMC could promote intrafibrillar mineralization, which is consistent with the TEM and SEM images.

Unlike the single-layer collagen fibrils, natural dentin is harder to mineralize because of the dense 3D collagen fibril network. Once dentin undergoes demineralization, the HAP reverts to a more basic form. When dentin is only partially demineralized, TMC can take advantage of calcium and phosphate ions as seed crystals to remineralize [30]. However, in the CAD area where the seed crystals are completely absent, although the collagen of demineralized dentin can be remineralized physiologically as it retains the intermolecular cross-linking and obvious cross-band characteristics seen in TEM images, the TMC can only make a disorderly deposition of calcium phosphate crystallization on the surface of demineralized collagen fiber without the guidance of NCP, resulting in different morphology and mechanical properties compared with natural dentin [31]. With the development of the MID concept, the ICCC (International Caries Consensus Collaboration) has recommended that caries should be selectively removed, by which means firm dentin and even soft dentin can be retained on the pulp wall to avoid pulp exposure. The ICCC emphasizes the preservation of CAD and considers it as an adhesive substrate for restoration [32]. Therefore, various attempts have been made using biomimetic materials to block the exposed dentin tubules and promote the adhesion interface, in order to achieve remineralization and restore demineralized dentin [30]. Clinically, the CAD depth is about a few hundred microns; therefore, we built an ACAD model through a pH-cycling protocol to simulate CAD in vitro [33] and thereby explored the effect of CMC on artificial carious dentin and its bond interface with resin.

The interfacial environment of the demineralized dentin matrix plays a determinant role in dentin bonding. However, the inner layer of demineralized dentin has changed in structure under the influence of caries-related bacteria [31]. Because of mineral deficiency, caries dentin lesions are reported to have high porosity and water content, a thicker hybrid layer, and lower adaptability than healthy dentin [34]. Therefore, improving the remineralization effect of the CAD that is penetrated by the adhesive and, thus, improving the internal adaptation and micromechanical properties [35,36] represent urgent problems to be solved in the clinical treatment of caries. In our study, the results of our dentin microhardness experiments suggested that our biomimetic mineralization system, in which CMC was used as a polymer, can mediate collagen fiber mineralization and enhance the dentin’s mechanical properties. This result can be explained via nanoleakage experiments, in which the dentin permeability of the BMD group showed a low permeability that is similar to normal dentin, suggesting that the collagen fiber gap was filled with minerals and that this improved the dentin’s hardness.

In addition to nanoleakage [37], thermocycling aging is also a useful method by which to evaluate bonding performance and quality at the interface. During the bonding process, the permeability of dentin affects the infiltration of the hydrophobic adhesive monomers, providing a favorable hydrophilic environment for the degradation of the hybrid layer, thereby decreasing the bond’s durability [38]. Thermocycling aging can assess the adhesion durability by imitating the changes in oral temperature caused by breathing and diet [39]. These changes in temperature can cause stress on the resin-dentin interface, owing to inconsistent volume shrinkage and expansion, which eventually leads to the formation of cracks [39]. CMC can induce intrafibrillar remineralization, which might result in a higher heat-resistant strain aggregation of the CMC-treated bonding interface, thus reducing crack formation. Consistent with our findings, the μTBS values did not change significantly after aging in the BMD groups but decreased significantly in the TMD group. At the same time, the incidence of adhesive layer fracture was significantly higher in the TMD group than in the BMD groups. Although the dentin hardness and μTBS of the biomimetic group were still lower than those of the natural dentin group, this may be due to the limited duration of mineralization.

Some researchers have tried to synthesize modified adhesive resins that release mineralized ions or mimic them to induce CAD biomimetic mineralization. However, because of the lack of chemical binding between the additives and the resin matrix, and because of the voids that will be left in the matrix after ion release, the mechanical properties and durability of the modified adhesive resin need to be evaluated [40,41]. Applying remineralizing agents before bonding can prevent the potential effects of adhesive additives and will hopefully develop into a new treatment for CAD [42].

As caries develop, the damage involved gradually spreads from the hard tissue to cause the decay of dental tissues close to the pulp. Hence, the restoration of deep caries includes not only the remineralization of hard tissues but also the restoration of the pulp–dentin complex. Therefore, restorative materials for deep caries are also important for the health of dental pulp tissues. Currently, indirect pulp capping or medical paste are often used in deep caries near the pulp, to promote remineralization and, thus, prevent further bacterial invasion and inflammation [43]. Since the ability of CMC to enable remineralization has been proven in this study, it can be used as a filler in dental materials near the pulp if the biocompatibility of CMC is acceptable. Our unpublished data explored the effects of CMC on the proliferation and adhesion of BMSCs via SEM, CLSM, and the Cell Counting Kit (CCK)-8. In the SEM images, the cells cultured on the BMC obviously presented polygon-like shapes with more pseudopods and stretched outwards, while those on the TMC were spindle-shaped, with fewer cellular pseudopodia. Flat cells were seen in the NMC. This phenomenon was further confirmed by CLSM, where BMSCs that were seeded on BMC scaffolds possessed more obvious pseudopodia compared with TMC. To explore the cytocompatibility of CMC, CCK-8 was used to compare the proliferation of BMSCs on various scaffolds. The CCK-8 assay showed that cell viability was the same within the first 24 h. After 48 and 72 h, cell viability in the BMC group was the highest. These results indicate that CMC had acceptable biocompatibility to some extent and could promote adhesion and proliferation of BMSCs. To better simulate the clinical and physiological characteristics, we will next explore the effects of CMC on dental pulp stem cells to provide evidence regarding the application of CMC in deep caries near the pulp.

The development of remineralization and the improvement of caries awareness have contributed to the evolution of caries treatment into a “minimally invasive” approach, advocating the preservation of the CAD, which can be remineralized using remineralizing fillers in dental materials, including adhesives and pulp-capping agents, to prevent the occurrence of pulp exposure. The ultimate goal is to preserve dental integrity by depositing a mineralized dentin matrix via pulp cells, establish a barrier to protect the pulp tissue itself, and reconstruct the homeostasis of the dentin–pulp complex [44]. Intrafibrillar mineralization can improve dentin microhardness as well as its bonding properties, but the ideal solution for deep caries should have a “double effect”, regenerating the defective dentin tissue while remineralizing the hard tissue. Hence, more in vivo and biocompatibility research should be carried out for clinical transformation.

## 5. Conclusions

Our study presented supporting data that CMC plays a significant role in intrafibrillar mineralization and, thus, improves the bonding effect. CMC-induced mineralization, as a new type of bonding strategy, may provide an MID treatment that preserves more hard tissues or even live pulp. Nonetheless, there is still much work to be done to bring CMC-induced mineralization into the clinic and to realize its full potential as a process-directing agent of biomineralization to repair CAD.

## Figures and Tables

**Table 1:** Vickers hardness measurements (MPa, mean ± SD) for each group.

	Vickers Hardness (MPa, Mean ± SD)
ND	75.1 ± 3.2 ^A^
DD	55.2 ± 2.9 ^B^
BMD	68.5 ± 2.5 ^C^
TMD	54.2 ± 2.8 ^B^
F value	252.735
*p*-value	<0.001

Different letters indicate statistically significant differences (*p* < 0.05).

**Table 2:** Microtensile bond strength (µTBS, MPa) values for each group.

	Before Thermal Cycling (Mean)	After Thermal Cycling (Mean)	F Value	*p*-Value
ND	35.9 (7.0) ^A1^	45.1 (6.7) ^A2^	18.33	<0.001
DD	26.4 (5.9) ^B1^	2.5 (2.2) ^B2^	57.026	<0.001
BMD	33.6 (4.3) ^A1^	26.7 (9.1) ^C1^	5.86	0.021
TMD	29.8 (8.3) ^A1^	1.9 (2.4) ^B2^	58.486	<0.001
F value	8.197	110.839		
*p*-value	<0.001	<0.001		

Values with identical numbers or letters imply no significant differences (*p* > 0.05) in each row or column, respectively.

## Data Availability

Not applicable.
